# Hydrocele Cured by One Tapping

**Published:** 1874-03

**Authors:** H. B. Lee

**Affiliations:** Atlanta, Ga.


### HYDROCELE CURED BY ONE TAPPING.

BY H. B. LEE, M.D., OF ATLANTA, GA.

T—— F—— T——, aged sixteen, came under my care, July 3, 1873. On examination I discovered a large hydrocele in the left tunica vaginalis; as the deformity was considerable, he was very anxious to have it removed, and willing to submit to any operation that would rid him of it.

The history of the case was quite simple; he had suffered some pain about four months before, after violent exereise on a pole; from that time he had noticed a swelling in the scrotum that had steadily increased till it reached the size I saw.

Owing to the extent of surface that I would have to inflame if I attempted the radical cure of such a large tumor, I determined to draw off the fluid and contract the scrotum, before it refilled, and not to inject any irritant into the sac till it had formed again.

I tapped him on the evening of the third of July, and drew off at least a pint of clear straw colored serum; prescribed lead lotions to the part and ordered perfect rest. On the fourth, to my surprise and disappointment, I found the sac had refilled by drainage from the surrounding cellular tissue, and was at least half as large as it had been at first.

I painted the scrotum with collodion, after contracting it as much as I could with cold and astringents, and told my patient he would have to submit to a further operation in about two weeks.

At the end of that time, on visiting him to perform the operation, I was pleased to find that absorption had commenced and the size of the tumor had been much reduced. In the faint hope that nature would perfect the cure, I directed frictions with iodine ointment and the use of a good suspensory bag. Since then the case has steadily progressed to a cure without any other treatment. On examining him yesterday, I could not detect the slightest trace of fluid in the tunic; it had resumed its normal size and appearance, and after such a length of time I have no hesitation in pronouncing the cure complete.

I claim no originality for this method of treatment, as the result was one I did not even hope for: all the text books on the subject, treating it as a very rare occurrence, and only met with in small hydroceles, most frequently those seen in tropical climates. My only object in publishing this case is to call the attention of the profession to its possibility, and to urge the employment of these simple and mild means before we subject our patients to the pain and danger of an injection into the cavity of the sac. It can do no harm, as it leaves your patient in a better condition for the radical operation, even if you fail to perfect the cure. If I save one poor fellow from the agony of an iodine injection I shall consider my time well spent.

*January* 20, 1874.

